# The human biological clock and aging—a comprehensive approach integrating reductionism, holism, and geromedicine for proactive healthspan strategies

**DOI:** 10.3389/fragi.2025.1658952

**Published:** 2025-08-18

**Authors:** Teow J. Phua

**Affiliations:** Molecular Medicine, NSW Health Pathology, John Hunter Hospital, Newcastle, NSW, Australia

**Keywords:** biological clock, biological aging, nitric oxide, hypoxia, chronic inflammation, healthspan, benign prostatic hyperplasia, prostate cancer

## Introduction

Currently, there is an array of established aging clocks that incorporate various factors such as epigenetic modifications, proteomic changes, inflammatory and immune pathways, neuroimaging techniques, and alterations related to the microbiome. These aging clocks possess significant therapeutic potential for alleviating the effects of chronic inflammation and associated diseases (Min et al., 2024).

Moreover, comprehensive multidisciplinary scoping reviews pertaining to the nitric oxide (NO)-mediated hypovascularity hypoxia hypothesis may shed light on the interconnected nature of biological aging clocks by integrating reductionist, holistic, and systems biology approaches. These longitudinal biological investigations are essential for elucidating persistent changes and big picture causal relationship patterns that underlie the primary biological mechanisms driving aging clocks (Phua, 2021; Phua, 2023; Phua, 2024).

### Reductionist, holistic and system biology

Reductionism is characterized by the breakdown of systems into their constituent parts to analyze individual functions, whereas holism underscores the interconnectedness and emergent properties of the system as a whole (Gatherer, 2010). As the landscape of scientific inquiry progresses, there is an increasing acknowledgment of the necessity for holistic approaches that recognize the importance of intricate interactions and emergent attributes (Delker and Mann, 2017).

Furthermore, the focus of systems-based geroscience is to accelerate research into the drivers of biological mechanisms that underlie aging, with the aim of developing improved clinical interventions for diseases and chronic conditions commonly experienced by the elderly population (Sierra, 2016; de Magalhães, 2024).

### Various medical approaches to the management of chronic diseases

Temporal medicine, which is inherently time-centric, examines the dynamics of disease progression over time, the effects of treatments at various stages of a patient’s illness, and the influence of intervention timing on patient health outcomes (Choudhary and Fränti, 2023; Rodenkirchen et al., 2025).

Integrative medicine combines established medical practices with evidence-based complementary therapies and lifestyle modifications to improve overall health and wellbeing. This holistic approach is particularly effective in preventing chronic diseases and managing existing conditions (Teut and Ortiz, 2021).

The future trajectory of precision geromedicine encompasses proactive, preventive, and interceptive strategies aimed at enhancing the healthspan (Amalaraj et al., 2025; Kroemer et al., 2025).

### The human biological aging clock

The concept of the biological aging clock posits that aging is an orderly and predictable process governed by intrinsic biological mechanisms, rather than merely a reflection of chronological age. This perspective indicates that aging adheres to a defined program, characterized by measurable markers that indicate an organism’s age and its proximity to mortality (Palmer, 2022).

In addition, the field of OMICS—which encompasses the comprehensive analysis and interpretation of multi-omics data (large datasets) representing the structure and function of biological systems at various levels—has significantly transformed our approaches to studying biological systems (Ahmed, 2022; Chen et al., 2023). This paradigm shift includes “top-down” methodologies, greatly influenced by the advancements in OMICS, integrated with “bottom-up” strategies, thereby providing a comprehensive toolkit to facilitate effective biological system investigations (Dai and Shen, 2022).

**FIGURE 1:**
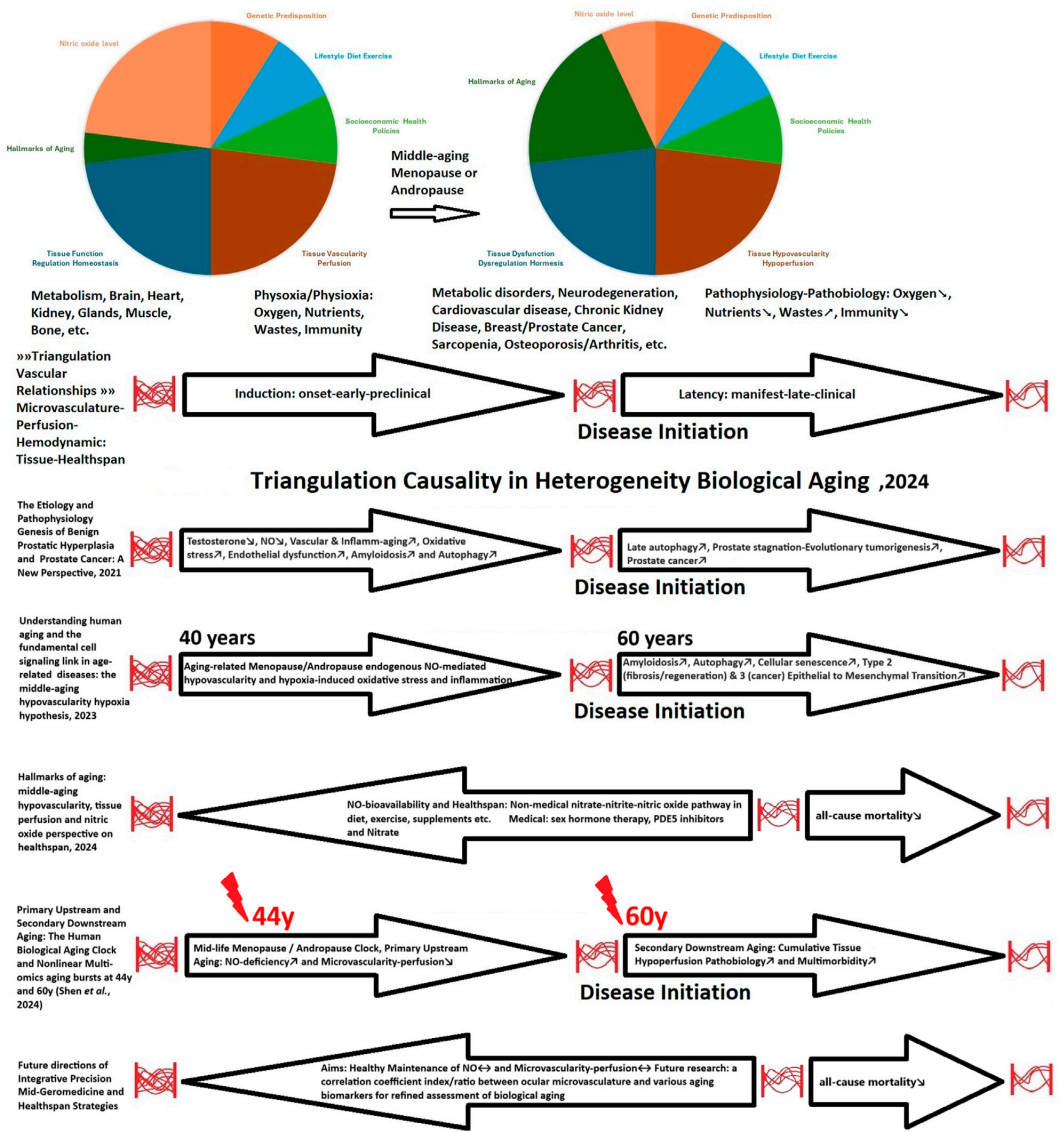
The Emergent Attributes in Human Biological Clock and Aging.

The emergent characteristics of multi-omics datasets highlight a deeper understanding of the predictable biological and cellular dynamics linked to the mid-life hormonal clock associated with menopause and andropause. Furthermore, they highlight the primary aging-related NO deficiency; a critical gasotransmitter, and micro-angiopathy; characterized by reducing microvascularity–perfusion function. Additionally, these attributes reflect the secondary cumulative pathobiology of tissue hypoperfusion, which involves hypoxia and chronic inflammation (Figure 1) (Phua, 2021; Phua, 2023; Phua, 2024).

### Healthspan strategies and precision middle-aged geromedicine: mid-geromedicine

The field of gerontology typically examines aging in individuals aged 65 and older (Lyu et al., 2024). It is important to note that the human biological aging clock is initiated in midlife, between the ages of 40 and 60 (Levine et al., 2016; Huang et al., 2025).

Consequently, public health prevention efforts should prioritize the middle-aged demographic as it presents the greatest opportunity for intervention at the upstream level. Woods et al. (2024); coined the term “precision middle-aged geromedicine—Mid-Geromedicine” (Figure 1).

The dynamic progression of aging (Ferrucci et al., 2020), particularly concerning early-stage (induction) microvascularity-perfusion networks, would demonstrate superior tissue functionality recovery in comparison to late-stage (latency) dysfunction, which is often compounded by cumulative pathobiological pathologies (Figure 1) (Phua, 2024). This transition dynamism (Yang et al., 2024) is linked to the positive outcomes associated with early sex hormone replacement therapies in alignment with the timing hypothesis (El Khoudary et al., 2020; Hodis and Mack, 2022), correlated with reductions in all-cause mortality during menopause (Arayici et al., 2024; Liu et al., 2024) and andropause (Jaiswal et al., 2024; Yeap et al., 2024), and increased production of NO (Jokela et al., 2003; Gur et al., 2020).

Furthermore, significant nonlinear multi-omics aging bursts are at observed at 44 and 60 years of age (Shen et al., 2024), coinciding with a decline in sex hormone levels and increasing inflammation (Ketchem et al., 2023; Allahverdiyeva et al., 2024; Lombardo et al., 2024) and the onset of increased multimorbidity in individuals aged 65 and older (Figure 1) (Divo et al., 2014; Song et al., 2023).

### Empirical perspective of endogenous nitric oxide (NO) as a gasotransmitter in biological aging

NO serves as a crucial empirical link between aging-related sex hormones and the vascular-aging process within the microvascularity–perfusion mechanistic network, a concept integral to the human biological aging clock and its beneficial health effects (Figure1) (Phua, 2023; 2024). Experimental reduction in sex hormone levels leads to the development of micro-angiopathy, while the supplementation of sex hormones reverses this condition, promoting micro-angiogenesis (Yura et al., 2020; Wang et al., 2022). NO actively promotes angiogenesis (Zhang et al., 2023). However, as individuals age, there is a notable decrease in overall NO production (Siervo et al., 2024), accompanied by reduced blood vessel density and altered dynamics of endothelial cell populations, within the aging endocrine system (Chen J. et al., 2021).

A paradoxical relationship exists between hypertension and capillary rarefaction (Frost et al., 2021), which characterizes microvascularity–perfusion aging, with NO acting as a principal driver in this process (da Silva et al., 2021; Bryan, 2022). This relationship is particularly evident in the context of chronic diseases (Bryan et al., 2023). In China, nearly one in four adults experiences multimorbidity, with hypertensive conditions frequently co-occurring with other health issues (Hu et al., 2024). Longitudinal accelerated aging is associated with the incidence of circulatory diseases (vascular-aging), related chronic conditions, and both all-cause and cause-specific mortality (Liu et al., 2023). Furthermore, studies involving human biopsies, autopsies, and imaging revealed a 32% reduction in median microvascular density (hypovascularity) (Querfeld et al., 2020).

In a state of health, the NO-cyclic 3′–5′ guanosine–monophosphate signaling pathway is vital for regulating smooth muscle tone, platelet function, cardiac contractility, renal operation, fluid homeostasis, and cellular growth (Mónica et al., 2016). Dysregulation of NO signaling is a common feature across various significant disorders, including cardiovascular disease, diabetes, and cancer (Lundberg and Weitzberg, 2022).

Research findings underscore the independent role of gasotransmitters in mediating age-related physiological and pathological processes, encompassing diabetes (van den Born et al., 2016), immune responses (Fagone et al., 2018), mitochondrial function (Hendriks et al., 2019), fibrotic diseases (Chen Y. et al., 2021), cardiovascular protection (Pagliaro et al., 2024), inflammatory edema (Coavoy-Sanchez et al., 2024), and age-associated oxidative stress (Munteanu et al., 2025).

In the modern era, individuals are increasingly seeking straightforward and natural methods to enhance their wellbeing and support both physical and mental health. This trend includes participation in activities at gyms and specialized wellness studios, as well as the adoption of healthy dietary practices. Many of these lifestyle interventions, often referred to as bio-hacking, are associated with the production of NO. Activities such as exercise (Arefirad et al., 2022) and exposure to natural sunlight (Hazell et al., 2022) play a crucial role in this process. Furthermore, gym supplements like L-arginine/-citrulline (Kiani et al., 2022), along with super-foods such as beetroots (Zamani et al., 2021) and pumpkin seeds (Akomolafe et al., 2025), are recognized for their potential to boost NO levels. Specialized wellness centers that offer hyperbaric oxygen therapy (Yamamoto et al., 2020), cold therapy (Wiecek et al., 2021), and light therapy (Kashiwagi et al., 2024) also contribute to this focus on health. Healthy dietary practices encompass consuming dietary nitrates as in following a Mediterranean diet (Mohajeri and Cicero, 2023). Conversely, the consumption of ultra-processed foods has been linked to a decline in NO production (Babalola et al., 2025). This also encompasses the effects of environmental and climatic factors on the processes of physiological aging (Zhang et al., 2024).

Moreover, two genetic variants GCH1 (Guo et al., 2025) and EPAS1 (Li C. et al., 2019) have been identified that enhance the adaptability of Tibetans residing at high altitudes in low-oxygen environments. The particular variant, GCH1, is associated with reduced expression levels, leading to increased NO production and enhanced oxygen delivery. Additionally, evidence indicates that these highland communities experience lower mortality rates from circulatory diseases and cancer (Wander et al., 2020; Burtscher et al., 2021).

### Integrative precision mid-geromedicine and prostate aging degeneration hypothesis

Integrative Precision Mid-Geromedicine leverages existing knowledge within its scientific domain to explore potential approaches for investigating pertinent issues or challenges, thereby providing deeper insights (McGregor and Frodsham, 2023).

The Prostate Aging Degeneration Hypothesis (Phua, 2021) predictable aging-related degeneration serves as a key illustration of mechanistic reasoning strategies aimed at addressing the underlying causes of symptoms (Flowers et al., 2023). Dysfunctions in prostate tissue remodeling result from alterations in smooth muscle function, prostate growth, enlargement, fibrosis, (Liu et al., 2019), and localized inflammation (Magri et al., 2019).

Benign prostatic hyperplasia (BPH/prostate enlargement) is a prevalent condition affecting approximately 50% of men over the age of 50, often accompanied by lower urinary tract symptoms (LUTS) (Haile et al., 2024), chronic pelvic pain syndrome (CPPS), chronic prostatitis (Pena et al., 2021), and incidence of prostate cancer exceeding 1 in 2 in men aged 65 and older (Rosario and Rosario, 2025).

The observation of abundant deposits of prostatic pro-inflammatory corpora amylacea (wasteosome-starch-like-bodies) (DuPre et al., 2018; Riba et al., 2022) is largely overlooked in conventional medicine. This neglect represents a research gap as the alternative explanations for BPH/enlargement-symptomology within a confined suprapubic space: the reduction in bladder capacity and urethral compression in LUTS and the pressure-related CPPS in the adjacent sensitive fascia (Gatt et al., 2025).

A hypoxic low-grade inflammation microenvironment is a characteristic feature of many tumors (Korbecki et al., 2021), largely resulting from inadequate vascular networks that fail to sufficiently supply oxygen (Alimoradi et al., 2016). To tackle this issue, the sex hormone bioavailability is essential for maintaining microvascularity–density health (Wang et al., 2022). However, testosterone therapy has been shown not to alleviate LUTS, but it does improve markers of prostatitis (anti-inflammatory) in men with BPH (Rastrelli et al., 2022). As such, a dual medical approach is warranted, one that addresses the aging-related NO-deficiency alongside complementary therapies such as regular prostatic drainage/massage to eliminate starch-like-wasteosomes (Sun and Bao, 2013), thereby mitigating prostatic size, pressure-related pain, and inflammation. This underscores the holistic dimension of integrative medicine and its potential role in the emerging field of Integrative-Precision-Mid-Geromedicine.

The eradication of localized chronic inflammation serves as a pivotal strategy in preventive medicine aimed at mitigating the risk of cervical cancer linked to human papillomavirus through vaccination initiatives (Jain et al., 2023). Similarly, this extends to the management of *Helicobacter pylori* infections and stomach cancer prevention (Li W.-Q. et al., 2019).

Importantly, testosterone and its reduced metabolites, 5α- and 5β-dihydrotestosterone, exert vasodilatory effects (Sánchez-Fernández et al., 2024). Testosterone has not been shown to increase the risk of prostate cancer (Siltari et al., 2023) and is anti-fibrotic (Chung et al., 2021). In fact, elevated testosterone levels are correlated with smaller prostate size (Xia et al., 2021). Conversely, the use of 5α-reductase inhibitors has been linked to an increased risk of acute coronary syndrome (Chou et al., 2015), sexual dysfunction (Corona et al., 2017), type-2 diabetes (Wei et al., 2019), age-related macular degeneration (Su et al., 2024), and suicide (Kim et al., 2025).

In a similar vein, phosphodiesterase-5 inhibitors restore NO-signaling (Lee et al., 2022) and exhibit anti-fibrotic (Li et al., 2024) and anti-inflammatory (Isidori et al., 2021) properties.

## Discussion

The future trajectories of Integrative-Precision-Mid-Geromedicine hold the promise of substantially diminishing the incidence of chronic diseases, guiding us toward paradigms of preventive geromedicine (Thomas et al., 2023; Hoenders et al., 2024). This approach empowers individuals to take charge of their healthspan strategies through proactive health education programs, ultimately leading to reduced healthcare costs and enhanced sustainability within healthcare systems.
